# Lactase persistence genotypes and malaria susceptibility in Fulani of Mali

**DOI:** 10.1186/1475-2875-10-9

**Published:** 2011-01-14

**Authors:** A Inkeri Lokki, Irma Järvelä, Elisabeth Israelsson, Bakary Maiga, Marita Troye-Blomberg, Amagana Dolo, Ogobara K Doumbo, Seppo Meri, Ville Holmberg

**Affiliations:** 1 Department of Bacteriology and Immunology, Haartman Institute, P.O. Box 21, FIN-00014, University of Helsinki, Finland; 2 Department of Medical Genetics, University of Helsinki, Helsinki, Finland; 3 Laboratory Services, Helsinki University Central Hospital, Helsinki, Finland; 4 Seattle Biomedical Research Institute, Seattle, USA; 5 Department of Immunology, Wenner-Gren Institute, Stockholm University, Stockholm, Sweden; 6 Division of Infectious Diseases, Department of Medicine, Helsinki University Central Hospital, Helsinki, Finland; 7 Department of Epidemiology of Parasitics Diseases, Malaria Research & Training Center, Faculty of Medicine, Pharmacy & Odonto Stomatology, University of Bamako, Bamako, Mali

## Abstract

**Background:**

Fulani are a widely spread African ethnic group characterized by lower susceptibility to *Plasmodium falciparum*, clinical malaria morbidity and higher rate of lactase persistence compared to sympatric tribes. Lactase non-persistence, often called lactose intolerance, is the normal condition where lactase activity in the intestinal wall declines after weaning. Lactase persistence, common in Europe, and in certain African people with traditions of raising cattle, is caused by polymorphisms in the enhancer region approximately 14 kb upstream of the lactase gene.

**Methods:**

To evaluate the relationship between malaria and lactase persistence genotypes, a 400 bp region surrounding the main European C/T_-13910 _polymorphism upstream of the lactase gene was sequenced. DNA samples used in the study originated from 162 Fulani and 79 Dogon individuals from Mali.

**Results:**

Among 79 Dogon only one heterozygote of the lactase enhancer polymorphism was detected, whereas all others were homozygous for the ancestral C allele. Among the Fulani, the main European polymorphism at locus C/T_-13910 _was by far the most common polymorphism, with an allele frequency of 37%. Three other single-nucleotide polymorphisms were found with allele frequencies of 3.7%, 1.9% and 0.6% each. The novel DNA polymorphism T/C_-13906 _was seen in six heterozygous Fulani. Among the Fulani with lactase non-persistence CC genotypes at the C/T_-13910 _locus, 24% had malaria parasites detectable by microscopy compared to 18% for lactase persistent genotypes (P = 0.29). Pooling the lactase enhancer polymorphisms to a common presumptive genotype gave 28% microscopy positives for non-persistent and 17% for others (P = 0.11).

**Conclusions:**

*Plasmodium falciparum *parasitaemia in asymptomatic Fulani is more common in individuals with lactase non-persistence genotypes, but this difference is not statistically significant. The potential immunoprotective properties of dietary cow milk as a reason for the partial malaria resistance of Fulani warrant further investigation.

## Background

During the last 10,000 years protection against malaria and lactase persistence have been two of the strongest selection forces shaping the human genome [1,2]. Protection against malaria has been important particularly in the holoendemic regions of tropical Africa. On the other hand, lactase persistence, allowing adults to drink milk, has been shown to be beneficial in all regions where the climate and environment have made it possible to herd dairy cattle [1]. In Western Africa, these two environmental forces of selection have converged as the nomadic Fulani people have settled in i.e. Mali, Guinea Conakry, Senegal, Gambia and Burkina Faso.

The Fulani are traditionally a nomadic, pastoralist, trading people, herding cattle, goats and sheep across the vast dry hinterlands of their domain, where they live in sympatry with the local agricultural populations. As lactose tolerance is generally rare among African ethnic groups, the Fulani with around 50% clinical lactose tolerance are an obvious exception among their sympatrics [3-5].

Lactase non-persistence (adult-type hypolactasia, "lactose intolerance") is a genetically determined normal trait where down-regulation of lactase activity occurs during childhood [6]. Mutations to tolerate milk lactose have occurred in human history enabling adults to use dairy products. Several single nucleotide polymorphisms have been identified to be associated with lactase persistence in the enhancer region residing 13.9 kb upstream of the lactase gene (*LCT*) at 2q21-22 [3,7-10].

Fulani people have been shown to be more resistant to malaria infection compared to other sympatric tribes in Western Africa [11]. Some differences in the immune responses have been shown, but still the complete explanation of this phenomenon has remained unsolved.

The hypothesis of this study postulates that the partial malaria resistance could be associated to the dietary habits of the Fulani. Fulani has a tradition of abundant milk consumption and are known to use more milk than sympatric tribes. In the study area the Dogon also use milk, but not as much as the Fulani. To test this hypothesis, the 400 bp region covering the known lactase persistence variants was screened and the association between lactase persistence genotypes and malaria indices in 162 Fulani and 79 Dogon individuals was analysed.

## Methods

### Study area and population

The field study was carried out in rural villages located in the district of Koro where the Fulani and Dogon live in sympatry. The study villages are located in Sahelian area in Mali, the area is situated halfway between Mopti and the country border to Burkina Faso, approximately 850 kilometre North-East of Bamako, the capital city of Mali. The Malaria Research & Training Center of the University of Bamako has established a permanent community based health centre in the village of Manteau. The area is mesoendemic for malaria and infections are mainly caused by *P. falciparum*. There is a dry season from October to May and a rainy season between June and September, leading to seasonal transmission of malaria, with most cases registered from July until December. Entomological assessment in September 2001 revealed the same entomological inoculation rate (EIR) of 0.38 infective bites/person/night in the two ethnic groups [12].

### Sample collection and clinical data

Blood samples from 162 Fulani and 79 Dogon individuals from an age and gender matched control cohort were collected in a cross-sectional study during the rainy season in 2005 as previously described [13,14]. The ethical committee of the Faculty of Medicine and Pharmacy, University of Bamako in Mali and the National Ethics Committee in Sweden approved the study. Informed consent of all participants or their parents was obtained. Power calculations of this sample size with 162 Fulani showed that an odds ratio larger than 2.7 would be significant.

### Malaria diagnosis

Thick blood smears were collected from all patients and stained with 3% Giemsa. Smears were read by microscopists. The number of parasites present per 300 leukocytes was counted and parasite density assuming mean leukocyte count was calculated. Study participants had several indices recorded including age, body temperature, occurrence of spleen enlargement as observed by palpation. Furthermore, haemoglobin levels and presence of *Plasmodium *genetic material was assessed from blood samples by polymerase chain reaction (PCR). As markers for repeated or chronic malaria infections, the genotype groups for presence of splenomegaly and anaemia (defined as hemoglobin less than 110 g/L) were compared. The material for this study was a cross-sectional cohort, and thereby the malaria positives cases represent asymptomatic parasitaemia, not clinical disease.

### Genotyping

DNA was extracted from filter papers using Chelex-100 method [13,15]. Briefly, three discs of peripheral blood soaked dry filter paper were pierced out and incubated in 0.5% saponin PBS (phosphate buffered saline) solution at 4°C overnight. The following day filter papers were washed in PBS at 4°C for 15-30 min, whereafter they were boiled in 5% Chelex-100 water solution for 15 min. Following vortexing for 30 s, samples were centrifuged at 10,000 rpm for 3 min. Supernatants containing DNA were then extracted and stored at -20°C.

The DNA fragment covering 400 bp around the original lactase persistence variant C/T_-13910 _in intron 13 of the *MCM6 *gene 13.9 kb upstream of the lactase gene [7] was amplified by PCR. Amplified fragments were sequenced. Primers 5'-CCTGGTTAATACCCACTGACCTA-3'for forward sequence and 5'-GTCACTTTGATATGATGAGAGCA-3'for reverse sequence were used in a reaction of 30 cycles using temperatures of 94°C for 30 s, 53°C for 30 s, and 72°C for 75 s following initial 10 min step at 94°C and proceeding final step of 10 min at 72°C. Amplitaq Gold (Applied Biosystems) enzyme was used in all reactions. PCR products were controlled for success of amplification on a 1.5% agarose gel containing ethidium bromide.

Samples were purified from excess primers by digestion using 2.5 U of Shrimp Alkaline Phosphatase USB and 5 U of Exonuclease I (New England Biolabs) in 37°C for 60 minutes preceding inactivation of 15 minutes at 80°C. Purified samples were prepared for sequencing using the BigDye 3.1 terminator (Applied Biosystems) as instructed by manufacturer. Sequencing reaction was as follows: initiating step of 96°C for 1 min, 25 cycles of 96°C for 10 sec, 53°C for 5 sec, 60°C for 4 min. Sequence samples were purified with Millipore Multiscreen plates (Millipore, USA) with Sephadex G-50 Superfine sepharose (Amersham Biosciences, Sweden), electrophoresis with an ABI 3730 DNA Analyzer (Applied Biosystems) and base calling using the Sequence Analysis 5.2 software (Applied Biosystems). The results were analysed by Sequencher 4.1.4 software (Gene Codes, USA).

### Statistics

Each of the malaria variables were grouped into two categories enabling application of chi square test of statistical significance for all variables. Power calculations for the sample size were done assuming a prevalence of *P. falciparum *parasitaemia of 25% in unexposed (lactase non-persistent). The calculations were done for a significance of 95% and a power of 80%. Statistical analyses were conducted using SPSS 15.0 for Windows (SPSS Inc.) and StatCalc EpiInfo version 6 (CDC). Mann-Whitney U-test was used for comparing parasite densities for different genotypes, which did not follow normal distribution.

## Results

**Figure 1:**
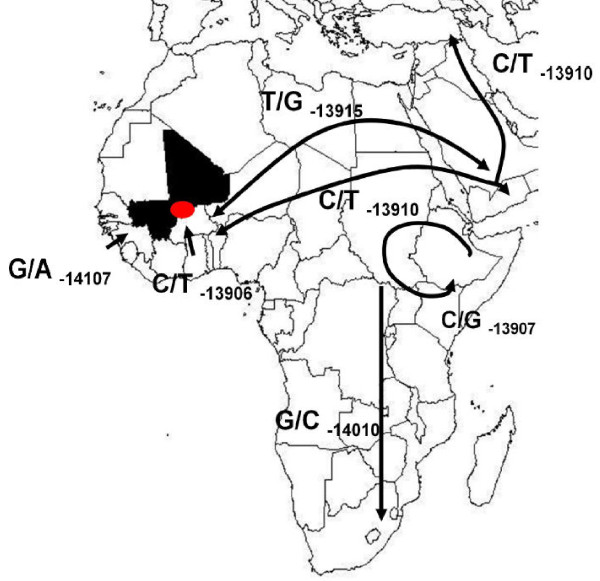
**African SNPs in the enhancer region of the *LCT *gene, in intron 13 of the *MCM6 *gene **[3,8,9,16,37]. Red colour points to the region of origin of samples in Mali (in black). Arrows represent suggested patterns of spread of the mutations. Without haplotype analyses between different populations, distinguishing original mutations from those that have spread between populations is not possible. Double arrow represents the fact that the original location of T/G_-13915 _cannot be determined, although it has been found in Arabic populations as well as in the Fulani of Mali (this study).

The enhancer region 14 kb upstream of the lactase gene, where the previously identified functional polymorphisms are located, was sequenced. In populations with European origin, C/T_-13910 _is the main causative mutation for lactase persistence [7]. In Africa, several polymorphisms in the enhancer region of the lactase gene have been suggested to be associated with lactase persistence (Figure 1).

In the sequenced region, four single nucleotide polymorphisms (SNP) were detected in the Fulani and Dogon under investigation (Table 1). C/T_-13910 _mutation, which is rare in most African populations, was found with a frequency of 37% in Fulani. Previously it has been detected with a minor allele frequency of 48% in Fulani from Sudan [4].

**Table 1:** The occurrence of four SNPs identified in this study in two ethnic tribes of Mali. N is the number of subjects with the given genotype.

		**C/T **_**-13910 **_**rs4988235**				
		No. with Genotype			Allele Frequency (%)	
Ethnicity	*N*	CC	CT	TT	C	T
Fulani	162	65	74	23	63.0	37.0
Dogon	79	78	1	0	99.4	0.6

		**G/A _-14107 _rs56150605**				
		**No. with Genotype**			**Allele Frequency (%)**	
		
Ethnicity	*N*	GG	GA	AA	G	A
Fulani	162	150	12	0	96.3	3.7
Dogon	79	79	0	0	100.0	0.0

		**T/C _-13906 _New SNP**				
		**No. with Genotype**			**Allele Frequency (%)**	
		
Ethnicity	*N*	TT	TC	CC	T	C
Fulani	162	156	6	0	98.1	1.9
Dogon	79	79	0	0	100.0	0.0

		**T/G _-13915 _rs41380347**				
		**No. with Genotype**			**Allele Frequency (%)**	
		
Ethnicity	*N*	TT	TG	GG	T	G
Fulani	162	160	2	0	99.4	0.6
Dogon	79	79	0	0	100.0	0.0

The mutation G/A_-14107 _previously reported in Ghana [16] had a minor allele frequency of 3.7% among Fulani. G/A_-13915 _is the founder lactase persistence variant among Urban Saudi population [8], and was seen in two heterozygote Fulani. One novel DNA polymorphism, C/T_-13906_, in the immediate proximity of the main European mutation, was found in six of the Fulani samples.

Of the 79 individuals belonging to the Dogon ethnic group, only one individual had a variant in the analysed region. This was a heterozygote of the globally most common mutation C/T at locus -13910.

To assess the hypothesised association between lactase persistence and malaria, the occurrence of the main functional C/T_-13910 _variant in Fulani was analysed (Table 2). At this locus, CC corresponds to lactase non-persistence, and both CT and TT to lactase persistence [7]. Among the Fulani with the CC genotype, 24% had malaria parasites present in the peripheral blood detectable with microscopy compared to 18% of those with genotypes CT/TT (P = 0.19, odds ratio 1.70, 95% confidence interval 0.72-4.03). The diagnostic PCR for *P. falciparum *was positive in 60% of subjects with lactase non-persistence (CC) and in 51% of lactase persistence (CT/TT) (P = 0.29, OR 1.41, 95% CI 0.71-2.80).

**Table 2:** *Plasmodium falciparum *parasitaemia in asymptomatic Fulani individuals grouped by observed genotype.

	Lactase Non-persistence	Lactase persistence	*P*-value	OR (95% CI)
Fulani samples	CC _-13910_	CT/TT _-13910_		
Malaria PCR positive, n (%)	39/65 (60%)	50/97 (51%)	0.29	1.41 (0.71-2.80)
Malaria microscopy positive, n (%)	15/65 (23%)	17/94* (18%)	0.44	1.36 (0.58-3.18)
**Fulani samples**	**Wild-type**	**Combined lactase persistence**		

Malaria PCR positive, n (%)	28/51 (55%)	61/111 (55%)	0.99	1.00 (0.49-2.05)
Malaria microscopy positive, n (%)	14/51 (28%)	18/108* (17%)	0.11	1.89 (0.79-4.51)

* Microscopy data missing for three individuals

Among the subjects with the CC genotype, splenomegaly was seen in 39% (25/65) and among the TC/TT genotypes in 35% (34/97) of the Fulani. Anemia was present in 56% (36/64) of CC individuals and in 58% (56/97) of TC/TT individuals. These differences were not statistically significant (p > 0.05).

Parasite densities of the Fulani positive for *P. falciparum *with microscopy were compared with different genotypes. The mean parasite density for the lactase non-persistance CC-13910 genotypes was 4,755 parasites/μL and for the CT/TT-13910 genotypes 2,584 parasites/μL (p = 0.56, Mann-Whitney U-test). The geometric means were 1,290 parasites/μL and 1,360 parasites/μL, respectively.

Assuming that all the detected SNPs in the lactase enhancer region would be functional, all patients with at least one polymorphic allele were pooled and then compared against the wild-type homozygotes (Table 2). The difference between at least one polymorphism in any of the loci and wild-type homozygotes was not significant either, even though a trend of more microscopy positive individuals in the lactase non-persistence group in comparison with the lactase persistence group was observed (p = 0.11, OR 1.89, 95% CI 0.79-4.51).

## Discussion

Several studies in Burkina Faso have indicated that people belonging to the Fulani tribe are less parasitized, have fewer clinical episodes of malaria, and higher levels of anti-malarial antibodies as compared to the sympatric ethnic groups Mossi and Rimabé, despite similar transmission intensity [17-21]. In Mali, Fulani have lower parasite density and are less affected by the disease compared to sympatric Dogon [22]. The more susceptible Dogon population seemed to respond to infections with pronounced splenomegaly, whereas Fulani have chronically enlarged spleens already functional for protection.

The reason for malaria resistance in Fulani is still unknown, but suggested mechanisms include a functional deficiency of regulatory T cells [21] and different antibody- and cytokine-mediated responses [23,24]. However, not much attention has been paid to the possible role of the different diets between Fulani and sympatric tribes. The Fulani diet is known to be mainly based on cow milk and also meat consumption is high. No detailed information on dietary habits of study subjects was available, although research on the subject is underway, so local observations and general knowledge on traditions of abundant milk consumption among Fulani in Western Africa were applied as sources of dietary data on population level. In a study in Nigeria, Fulani were described to get 28-29% of their total energy intake from milk products, which would correspond to about 700 grams of milk daily [25]. This amount of milk would probably cause symptoms for lactase non-persistence adults. Thus it is likely that Fulani in Mali with lactase persistence genotypes consume more milk than those with non-persistence genotypes.

The hypothesis of the present study postulates that the increased ability to drink milk in individuals with lactase persistence genotypes may offer protection against malaria infection. In human evolution, lactase activity has been maintained by mutations in the enhancer region of the lactase gene in those populations which herd livestock and will thus benefit substantially from the ability to use dairy products as part of the adults' diet. Several such mutations have enriched over time in isolated livestock herding populations, although smaller than expected differences among these genetically diverse populations have been reported [26]. In populations with European origin, C/T_-13910 _is the main causative mutation for lactase persistence [7]. In Africa several polymorphisms in the enhancer region of the lactase gene have been suggested to be associated with lactase persistence (Figure 1). Among the 162 Fulani genotyped, the major Caucasian mutation C/T_-13910 _was by far the most common polymorphism with an allele frequency of 37%. This means that 60% of the Fulani carry the lactase persistence genotype. Possibly the above described sympatry with other ethnic groups is responsible for the lower minor allele frequency found in this study than the one described by Enattah *et al *[4]. Three other SNPs in the region were found. They were found as heterozygotes in twelve, six and two subjects each. Among the 79 Dogon samples tested only one heterozygote of a lactase enhancer polymorphism, the main European mutation, was detected.

Results show that *P. falciparum *parasitaemia in asymptomatic Fulani is more common in individuals with lactase non-persistence genotypes. However, this finding was not statistically significant as this study did not have power to detect a difference with odds ratio smaller than 2.7. The rarity of the SNPs among Dogon and among other non-pastoralist populations in Africa further supports the hypothesis. Asymptomatic parasitaemia was used as the main phenotype, assessment of the suggested protective effects of lactase persistence on clinical malaria episodes, severe malaria or malaria mortality was not applicable. The novel T/C_-13906 _polymorphism detected in this study cannot be claimed to be functional without lactose tolerance test or intestinal biopsy on individuals with this variant. However, the location only 4 bp from the main mutation supports the possibility of functionality. In addition, the lack of polymorphisms in the Dogon subjects suggests that the variability in the Fulani lactase enhancer region is caused by ongoing selection for which functionality is a prerequisite.

The potential protective role of milk consumption in malaria infections has been debated for more than half a century. Studies in the 1950s indicated that a cow milk diet has a suppressive effect on malaria infections in rats, mice and monkeys [27]. The suppression of the normal replication of the parasites was suggested to be caused mainly by the deficiency of *p*-aminobenzoic acid (PABA), as milk is lacking PABA in contrast to normal diets [28]. *Plasmodium *species can synthesize PABA *de novo*, but probably not in sufficient amounts to survive without dietary intake by the host [29].

Infants under six months of age have a lower incidence of severe malaria than older children, which mainly has been explained by maternal antibodies acquired through breast-feeding. Deficiency of PABA might be another reason, especially for children exclusively breast fed. Additionally, there might be other still unidentified anti-malarial properties of milk, as it consists of numerous probiotics, innate immunity components (i.e. mannose binding-lectin, properdin, interferons), cytokines, chemokines and anti-inflammatory factors [30].

The relationship between nutrition and malaria seems to be complex. In the 1990s it was suggested that lactase non-persistence would have been selected by malaria, like beta-thalassaemia and glucose 6-phosphate dehydrogenase deficiency [31,32]. This hypothesis was soon rejected, as it was based on the false assumption that the lactase persistence trait would be the wild-type allele [33,34]. Reduced malaria morbidity in malnourished children and malaria outbreaks following refeeding after famines in Africa has been reported [35]. However, Fulani with diets consisting mainly of milk, did not have increased incidences of severe malaria after refeeding [36].

The potential protective role against malaria infection achieved by abundant milk consumption, allowed by lactase persistence genotypes, could have many explanations. First, milk might provide a generally increased nutritional status being rich in energy, proteins and fatty acids. Secondly, milk consists of a large number of immunomodulating components. Third, a diet dominated by dairy products might lead to a relative PABA deficiency, protecting from malaria. Additionally, there might be still unrecognized factors in milk providing protection against malaria and other infections.

## Conclusions

*Plasmodium falciparum *parasitaemia in asymptomatic Fulani is more common in individuals with lactase non-persistence genotypes, but this difference is not statistically significant. A novel polymorphism in the *LCT *enhancer region was discovered with potential functional importance. The lack of *LCT *enhancer region polymorphisms in the sympatric Dogon suggests positive selection in the Fulani who have access to dietary milk. The potential immunoprotective properties of dietary cow milk as a reason for the partial malaria resistance of Fulani warrant further investigation.

## Competing interests

The authors declare that they have no competing interests.

## Authors' contributions

IL extracted the DNA from blood samples stored in Stockholm University and performed sequencing and analysis of sequence data in University of Helsinki. VH introduced the original idea for study and drafted the manuscript together with IL. IJ supervised laboratory work in University of Helsinki and provided expertise in lactase genetics during writing of the manuscript. SM provided insight to the discussion of immunologic properties of milk and infection biology of malaria. EI and MTB supervised laboratory work in Stockholm University. Furthermore, MTB provided the blood samples while EI participated in the collection of the samples. BM, AD and OKD provided expertise of the research material, described the study population, and collaborated in organizing the field work for collecting the data. All authors read and approved of the final manuscript.
